# Saphenous nerve block as a diagnosis tool for chronic postsurgical pain of the left medial calf

**DOI:** 10.1002/ccr3.1373

**Published:** 2018-01-04

**Authors:** Dan Sebastian Dîrzu, Theodor Boţ, Constantin Ciuce

**Affiliations:** ^1^ University of Medicine and Pharmacy “Iuliu Hațieganu” Cluj Napoca Cluj Napoca Romania; ^2^ Emergency County Hospital Cluj Napoca Cluj Napoca Romania

**Keywords:** Chronic pain, diagnostic nerve block, hydro‐dissection, saphenous nerve block, ultrasound‐guided nerve blocks

## Abstract

Ultrasound‐guided saphenous nerve block above the knee may be a valuable diagnosis tool in patient presenting chronic pain of the calf.

## Question

What diagnosis information can an ultrasound‐guided saphenous nerve block provide when performed for chronic pain relief of the medial calf?

## Answer

A 41‐year‐old woman presented with chronic pain in the left saphenous nerve distribution after multiple reconstructive surgeries. She reported rest pain of 80 mm on visual analog pain scale and 100 mm while standing. Ultrasound‐guided saphenous block above the knee was described for chronic pain of the knee 1, 2. Because of the pain distribution, we indicated and performed the procedure. The movie (Video S1) shows saphenous nerve entrapped in a fibrous scar produced by surgical incision and the hydro‐dissection performed with 8 mL Ropivacaine 1% and 2 mL Betamethasone 5+2 mg/mL. Patient was completely pain free for 48 h but pain reappeared weeks later. After a second injection with same dynamic of the pain, surgical neurolysis was realized with good long‐term results

## Conflict of Interest

Theodor Boț and Constantin Ciuce have no conflict of interest to disclose. Dan Sebastian Dîrzu acts as Senior Editor for CCRJ.

## Authorship

DSD, TB, and CC: had the same contribution in documenting the case, indication for the procedure, follow‐up of the patient, and writing the manuscript. DSD: performed the procedure. TB: assisted performing the procedure.

## Supporting information


**Video S1**. Ultrasound‐guided saphenous nerve block hydro‐dissection.
